# Impaired pulmonary vasomotor control in exercising swine with multiple comorbidities

**DOI:** 10.1007/s00395-021-00891-7

**Published:** 2021-09-12

**Authors:** Jens van de Wouw, Jarno J. Steenhorst, Oana Sorop, Ruben W. A. van Drie, Piotr A. Wielopolski, Alex Kleinjan, Alexander Hirsch, Dirk J. Duncker, Daphne Merkus

**Affiliations:** 1 Division of Experimental Cardiology, Department of Cardiology, Erasmus MC, University Medical Center Rotterdam, PO Box 2040, 3000 CA Rotterdam, The Netherlands; 2 Department of Radiology and Nuclear Medicine, Erasmus MC, University Medical Center Rotterdam, Rotterdam, The Netherlands; 3 Department of Pulmonary Medicine, Erasmus MC, University Medical Center Rotterdam, Rotterdam, The Netherlands; 4 Department of Cardiology, Erasmus MC, University Medical Center Rotterdam, Rotterdam, The Netherlands; 5 Institute for Surgical Research, Walter Brendel Center of Experimental Medicine (WBex), University Clinic Munich, LMU Munich, Munich, Germany; 6 German Center for Cardiovascular Research, Partner Site Munich, Munich Heart Alliance, Munich, Germany

**Keywords:** Diabetes mellitus, Chronic kidney disease, Pulmonary hypertension, Endothelin, Nitric oxide

## Abstract

Pulmonary hypertension is common in heart failure with preserved ejection fraction (HFpEF). Here, we tested the hypothesis that comorbidities [diabetes mellitus (DM, streptozotocin), hypercholesterolemia (HC, high-fat diet) and chronic kidney disease (CKD, renal microembolization)] directly impair pulmonary vasomotor control in a DM + HC + CKD swine model. 6 months after induction of DM + HC + CKD, pulmonary arterial pressure was similar in chronically instrumented female DM + HC + CKD (*n* = 19) and Healthy swine (*n* = 18). However, cardiac output was lower both at rest and during exercise, implying an elevated pulmonary vascular resistance (PVR) in DM + HC + CKD swine (153 ± 10 vs. 122 ± 9 mmHg∙L^−1^∙min∙kg). Phosphodiesterase 5 inhibition and endothelin receptor antagonism decreased PVR in DM + HC + CKD (− 12 ± 12 and − 22 ± 7 mmHg∙L^−1^∙min∙kg) but not in Healthy swine (− 1 ± 12 and 2 ± 14 mmHg∙L^−1^∙min∙kg), indicating increased vasoconstrictor influences of phosphodiesterase 5 and endothelin. Inhibition of nitric oxide synthase produced pulmonary vasoconstriction that was similar in Healthy and DM + HC + CKD swine, but unmasked a pulmonary vasodilator effect of endothelin receptor antagonism in Healthy (− 56 ± 26 mmHg∙L^−1^∙min∙kg), whereas it failed to significantly decrease PVR in DM + HC + CKD, indicating loss of nitric oxide mediated inhibition of endothelin in DM + HC + CKD. Scavenging of reactive oxygen species (ROS) had no effect on PVR in either Healthy or DM + HC + CKD swine. Cardiovascular magnetic resonance imaging, under anesthesia, showed no right ventricular changes. Finally, despite an increased contribution of endogenous nitric oxide to vasomotor tone regulation in the systemic vasculature, systemic vascular resistance at rest was higher in DM + HC + CKD compared to Healthy swine (824 ± 41 vs. 698 ± 35 mmHg∙L^−1^∙min∙kg). ROS scavenging induced systemic vasodilation in DM + HC + CKD, but not Healthy swine. In conclusion, common comorbidities directly alter pulmonary vascular control, by enhanced PDE5 and endothelin-mediated vasoconstrictor influences, well before overt left ventricular backward failure or pulmonary hypertension develop.

**Supplementary Information:**

The online version contains supplementary material available at 10.1007/s00395-021-00891-7.

## Introduction

Pulmonary vascular disease (PVD) is prevalent in a large proportion of patients with heart failure with preserved ejection fraction (HFpEF); about one out of four patients meet the criteria of overt pulmonary hypertension (PH) in this population (mean pulmonary arterial (PA) pressure > 25 mmHg at rest), which is associated with increased mortality and cardiac hospitalization [31, 32, 60]. It is increasingly recognized that even a slightly elevated PA pressure—above 19 mmHg—is a strong predictor of increased hospitalizations and higher mortality [11, 35], underlining the importance of early recognition and treatment of PVD. Although the early symptoms of PVD can be subtle at rest, exercise is able to reveal PVD in an early stage in patients with cardiopulmonary diseases, including HFpEF [18, 21, 31, 34].

Common comorbidities for cardiovascular disease, including diabetes mellitus (DM), hypercholesterolemia (HC), chronic kidney disease (CKD), are independently—but especially in combination—well-known risk factors for the development of HFpEF [10, 43]. The current paradigm of HFpEF implies a crucial role for coronary microvascular dysfunction, due to a systemic pro-inflammatory state, in its development [19, 43]. Yet, the pro-inflammatory state is not restricted to the coronary microvasculature and hence, other vascular beds—not only the systemic, but also the pulmonary vasculature—may also be directly affected [48]. Indeed, PH is highly prevalent in patients with CKD [6, 51]. Thus, contrary to the current belief that PH progresses from early post-capillary PH due to elevated left atrial pressures, to combined pre- and post-capillary PH with pulmonary vascular changes, a direct detrimental effect of the systemic pro-inflammatory state on the pulmonary vasculature may also be present, suggesting that PVD could develop prior to overt HFpEF-PH in a subgroup of patients. This pro-inflammatory state may induce pulmonary endothelial dysfunction reflected by an imbalance between the influences of the vasoconstrictor endothelin (ET) and the vasodilator nitric oxide (NO) on control of pulmonary vascular tone [41].

In light of these considerations, we aimed to investigate the effects of a chronic pro-inflammatory state, induced by 5 months of DM, HC and CKD, on pulmonary microvascular and right ventricular (RV) structure and function. The effects of DM, HC and CKD on the systemic and pulmonary vascular function were assessed with a focus on endothelial dysfunction, and the balance between NO and ET, in chronically instrumented female swine with DM + HC + CKD at rest and during graded treadmill exercise [50, 55].

## Materials and methods

### Animals

All animal experiments were approved by the Animal Care Committee at the Erasmus University Medical Center (Rotterdam, The Netherlands) and in accordance with the “Guiding Principles in the Care and Use of Laboratory Animals” as approved by the National Research Council of the National Academies. 19 female Yorkshire × landrace swine (24 ± 1 kg) were included in the experimental group (DM + HC + CKD) while 18 healthy female Yorkshire × landrace swine of similar age and weight were used as controls (Healthy).

### Induction of risk factors

The induction of risk factors in the DM + HC + CKD group has been described in detail elsewhere [50]. Briefly, DM was produced by injection of streptozotocin (AdipoGen Life Sciences, San Diego, CA, USA) in a dose of 50 mg∙kg^−1^ per day i.v. on three consecutive days. The severity and stability of DM was monitored bi-weekly by measurements of blood glucose and ketone levels.

2 weeks after DM induction, animals were sedated with intramuscular injection of a cocktail of Zoletil (tiletamine/zolazepam; 5 mg∙kg^−1^), Sedazine (xylazine; 2.25 mg∙kg^−1^) and atropine (2 mg) and artificially ventilated (O_2_ and N_2_ [1:2 vol/vol], to which 1–2% (vol/vol) isoflurane was added for anesthesia). CKD was produced by microembolization of the global right kidney as well as the lower pole of the left kidney. For this purpose, the renal arteries were catheterized under fluoroscopy guidance (right renal artery and selective catheterization of the artery perfusing the left lower renal pole) with a Swan-Ganz catheter, inserted through a 9 F sheath in the right common carotid artery. Following inflation of the balloon to prevent back-flow into the aorta, 75 mg of polyethylene microspheres with a diameter of 38–42 μm (Cospheric, Santa Barbara, CA, USA) were infused in each kidney. The wound was closed and the animals were allowed to recover.

1 week after CKD induction, a high-fat and high-sugar diet containing 10% sucrose, 15% fructose, 25% saturated fats and 1% cholesterol (Research Diets Services BV, Wijk bij Duurstede, The Netherlands) supplemented with sodium chloride (20 g per day) was gradually introduced. The Healthy group continued to receive regular swine-chow. Animals were housed in pairs but were fed separately and had ad libitum access to drinking water.

The animals were divided into two groups; one group (12 DM + HC + CKD and 12 Healthy) was chronically instrumented 5 months following induction of the risk factors, as described elsewhere [9], and renal function measurements and exercise experiments were performed 1–3 weeks later. The second group (7 DM + HC + CKD and 6 Healthy) underwent cardiovascular magnetic resonance imaging (CMR) using a 1.5-T scanner 6 months after induction of the risk factors and sacrificed afterwards.

### Instrumentation

As mentioned above, one group of animals underwent chronic instrumentation after 5 months and was terminated 1 month later. For chronic instrumentation, swine were sedated, intubated and anesthetized as described above. As described elsewhere [9], a thoracotomy was performed in the fourth left intercostal space under sterile conditions. After opening of the pericardium, fluid-filled polyvinylchloride catheters (Braun Medical Inc., Bethlehem, PA, USA) were inserted into the pulmonary artery (2 ×), the aortic arch, the left atrium (2 ×) and the right ventricle to allow hemodynamic measurements and extraction of blood samples. A transit-time flow probe (Transonic Systems Inc., Ithaca, NY, USA) was placed around the ascending aorta for measurement of cardiac output (CO) [9]. Electrical wires and catheters were tunneled subcutaneously to exit at the back and protected with a vest. Then, the chest was closed in layers, and animals were allowed to recover, receiving analgesia (0.3 mg buprenorphine i.m. once) and a slow-release fentanyl patch (50 μg h^−1^) and antibiotic prophylaxis (25 mg∙kg^−1^ amoxicillin i.v.) for 7 days. All catheters were flushed daily with heparinized saline (1000–5000 IU ml^−1^ saline) to prevent the formation of blood clots and to ensure catheter patency [9].

### Exercise experiments

After 1 week of recovery, exercise experiments were conducted on a motor-driven treadmill. Briefly, resting hemodynamic measurements, blood samples and rectal temperature were obtained with swine standing quietly on the treadmill. Subsequently, all swine were subjected to a three-stage incremental treadmill exercise protocol (2–4 km∙h^−1^ at 0% inclination, 3 min per speed). Hemodynamic variables, consisting of heart rate, cardiac output, aortic pressure, PA pressure, left atrial pressure and RV pressure were continuously recorded digitally on a Codas workstation (ATCODAS, Dataq Instruments, Akron, OH, USA) with blood samples collected at rest and during the final 30 s of each 3-min exercise stage when steady-state hemodynamics had been achieved. Blood samples were analyzed for PO_2_, PCO_2_, pH, O_2_ saturation and hemoglobin concentration (ABL-800, Radiometer, Copenhagen, Denmark).

After 60 min of rest or on a following day the same exercise protocol was conducted while nitric oxide synthase (NOS), phosphodiesterase 5 (PDE5), endothelin receptor A and B (ET_A_/ET_B_), or reactive oxygen species (ROS) were inhibited. An overview of the number of animals in the different protocols, and the number of overlapping animals between protocols is given in Table 1. NOS inhibition (NOSi) was achieved by 20 mg∙kg^−1^ i.v. infusion of Nω-nitro-l-arginine (l-NNA, Sigma–Aldrich, Saint Louis, MO, USA), 15 min after l-NNA infusion, samples were obtained at rest and the exercise protocol was started. PDE5 inhibition (PDE5i) was achieved by administration of 10 mg Sildenafil (Revatio, Pfizer Inc, New York, NY, USA); 5 min after complete infusion resting samples were obtained and the exercise protocol was started. Endothelin receptor inhibition (ET_A + B_i) was achieved by infusion of the mixed ET_A_/ET_B_ receptor blocker Tezosentan (a gift from Actelion Pharmaceuticals Ltd, Allschwil, Switzerland, dosage: bolus of 300 µg∙kg^−1^∙min^−1^ over 10 min i.v. followed by continuous infusion of 100 μg∙kg^−1^∙min^−1^ i.v.) during the exercise protocol. Combined NOSi and ET_A + B_i was achieved by inhibition of NOS as described above, followed 15 min later, by ET_A + B_i using the same protocol as described above. ROS scavenging was achieved by continuous infusion of 1 mg∙kg^−1^∙min^−1^ of free radical scavenger N-(2-mercaptopropionyl)glycine (MPG, Sigma–Aldrich, Zwijndrecht, The Netherlands) and 30 mg∙kg^−1^ i.v. infusion of superoxide dismutase mimetic 4-hydroxy-2,2,6,6-tetramethylpiperidine-*N*-oxyl (Tempol, Sigma–Aldrich, Zwijndrecht, The Netherlands). After 10 min of infusion, samples were obtained at rest and exercise protocol was started.

**Table 1:** Schematic overview of swine used in various exercise protocols, as well as overlap between groups

Experiment	Con	ET_A + B_i	NOSi	NOSi/ET_A + B_i	PDE5i	ROSi
Con	**Diseased 12** **Healthy 11**	Healthy 6	Healthy 7	Healthy 5	Healthy 6	Healthy 8
ET_A + B_i	Diseased 6	**Diseased 6** **Healthy 6**	Healthy 4	Healthy 5	Healthy 6	Healthy 5
NOSi	Diseased 6	Diseased 4	**Diseased 6** **Healthy 7**	Healthy 5	Healthy 5	Healthy 4
NOSi/ET_A+B_i	Diseased 4	Diseased 4	Diseased 3	**Diseased 4** **Healthy 6**	Healthy 6	Healthy 6
PDE5i	Diseased 7	Diseased 4	Diseased 3	Diseased 3	**Diseased 7** **Healthy 7**	Healthy 6
ROSi	Diseased 8	Diseased 5	Diseased 6	Diseased 3	Diseased 4	**Diseased 8** **Healthy 8**

Numbers in bold denote the total numbers in a protocol, diseased reflect animal with DM + HC + CKD

*Con* control exercise experiment without pharmacological intervention, *NOSi* nitric oxide inhibition, *ET*_*A + B*_*i* endothelin receptor A and B antagonism, *PDE5i* phosphodiesterase 5 inhibition, *ROSi* scavenging of reactive oxygen species

### Cardiovascular magnetic resonance imaging acquisition and analysis

The second group of animals (DM + HC + CKD *n* = 7 and Healthy *n* = 6) underwent CMR 6 months after induction of the comorbidities. The animals were sedated with an intramuscular injection of Zoletil (tiletamine/zolazepam; 5 mg∙kg^−1^), Sedazine (xylazine; 2.25 mg∙kg^−1^) and atropine (2 mg), anesthetized with pentobarbital (bolus of 20 mg∙kg^−1^ followed by 10 mg∙kg^−1^∙h^−1^ i.v. during CMR) and sacrificed afterwards. Swine were placed in lateral position and mechanical ventilation and breath-holds were performed using a mobile ventilator (Carina, Dräger Medical, Best, The Netherlands). CMR was performed on a 1.5-T clinical scanner with a dedicated 32-channel phased-array surface coil (Discovery MR450, GE Healthcare, Milwaukee, Wisconsin, USA). The imaging protocol consisted of retrospectively ECG‐gated 2D balanced Steady‐State Free Precession cine imaging with breath‐holding (FIESTA, GE Healthcare acronym). Standard long‐axis and short‐axis images with full left ventricle and RV coverage were obtained. Typical scan parameters were slice thickness 6.0 mm, slice gap 0 mm, TR/TE 3.3/1.4 ms, flip angle 60°, NEX 2, field of view 288 × 360 mm, acquired matrix 160 × 192, and number of reconstructed phases 24 per cardiac cycle. To assess RV volumes, endocardial contours were drawn manually on end‐diastolic and end‐systolic short axis cine images. Volumes were measured and ejection fraction was calculated. All volumes were indexed for body weight. RV global longitudinal strain (GLS) was measured using the 4-chamber longitudinal axis by manually drawing endocardial contours during end-diastole and end-systole of the RV with subsequent automatic tracking during the entire cardiac cycle. The analyses were done with QMass (version 8.1) and QStrain (version 2.0) analytical software from Medis Medical Imaging Systems BV (Leiden, The Netherlands).

### Termination

At sacrifice the animals were sedated with intravenous infusion of Zoletil (tiletamine/zolazepam; 5 mg∙kg^−1^), Sedazine (xylazine; 2.25 mg∙kg^−1^) and atropine (2 mg) and anesthetized with pentobarbital (bolus of 20 mg∙kg^−1^ followed by 10 mg∙kg^−1^∙h^−1^ i.v.). Subsequently, a sternotomy was performed and ventricular fibrillation was induced using a 9 V battery, and immediately the heart and lungs were excised and stored for later analysis.

### Plasma measurements

Fasting arterial blood samples were obtained at instrumentation (5 months follow-up) or immediately after CMR (6 months follow-up) for determination of plasma glucose, triglycerides, total cholesterol, low-density lipoprotein (LDL), high-density lipoprotein (HDL) and creatinine. Arterial plasma concentrations of tumor necrosis factor alpha (TNF-α, R&D Systems Inc., Minneapolis, MN, USA) and endothelin-1 (ET-1, Enzo Life Sciences International Inc., Farmingdale, NY, USA) were determined using ELISA kits, according to the manufacturer’s protocol. NO metabolites, nitrite and nitrate (NO_2_^−^ + NO_3_^−^), were determined using a colorimetric Griess reaction assay (BioVision Inc., Milpitas, CA, USA). The glomerular filtration rate (GFR) was measured in chronically instrumented animals using continuous inulin infusion (19 mg∙min^−1^, Inutest Fresenius Pharma, Austria, GmbH) and plasma sampling at rest. Three consecutive 20 min inulin clearance periods were averaged.

### Histological measurements

Samples of the lung and RV wall were excised, fixated in 4% buffered formaldehyde and embedded in paraffin for histological analyses. RV wall sections (4.5 μm thick) were stained for quantification of myocardial collagen deposition, myocyte size and capillary density. Six to eight fields were examined in the subendocardial half of each slide, at 20 × magnification. Interstitial collagen deposition was assessed using picrosirius red staining, with perivascular collagen deposition being excluded from the analysis. A polarization filter differentiated between collagen type I and III fibers [59]. The areas occupied by the different types of collagen fibers were measured and expressed as a percentage of the myocardial area. Cross-sectional areas of cardiomyocytes with clearly visible nuclei were measured for each slide, using a Gomori silver stain. Capillary density per mm^2^ myocardial area was quantified using an endothelial cell staining with biotin-labeled lectin (lectin 1/100 in 1% bovine serum albumin in PBS, Sigma–Aldrich, Zwijndrecht, The Netherlands). All vessels smaller than 10 μm in diameter and without vascular smooth muscle cells were counted. Capillary density was divided by the number of cardiomyocytes per mm^2^, quantified in Gomori stained sections, to calculate capillary-to-fiber ratios. All measurements were performed using a microscopy image analysis system (Impak C, Clemex Vision Image analysis system, Clemex Technologies, Quebec, Canada) and by a blinded observer.

After excision of the lungs, the accessory lobe was inflated and perfusion-fixated with 4% buffered formaldehyde at a pressure of 25 cm H_2_O and embedded in paraffin. Vascular structure was assessed in pressure fixated lung tissue using a Resorcin–Fuchsin–Van Gieson’s (RF) staining was performed to discriminate the internal and external elastic lamina of small pulmonary arteries. Using the Hamamatsu NanoZoomer Digital Pathology (NDP) slide scanner (Hamamatsu Nanozoomer 2.0HT, Hamamatsu Photonics K.K., Hamamatsu City, Japan), whole section images were obtained. Morphometric measurements of pulmonary small arteries were performed using NDP viewer (Hamamatsu) by a blinded observer. Both internal and external elastic lamina areas were measured and assuming circularity of the vessels, inner and outer radius were calculated as *r* = √(area/π). Wall‐to‐lumen ratio was calculated as (outer − inner radius)/inner radius, and relative lumen area as inner/outer area. To ensure that pulmonary veins were excluded from analysis, vessels in close proximity to the intersegmental septae were excluded from analysis. Only transversely cut vessels with an outer diameter of 30–120 μm were analyzed.

Endothelial cells were labelled with lectin (1:100 in 1% bovine serum albumin in TBS, Sigma–Aldrich, Saint Louis, MO, USA) and smooth muscle cells with a monoclonal mouse anti-human smooth muscle actin (SMA, 1:500 diluted with 1% bovine serum albumin in TBS, Agilent Technologies, Santa Clara, CA, USA) using a double stain system (DAKO EnVision G, Agilent Technologies). Following image digitization using the Hamamatsu NanoZoomer Digital Pathology (NDP) slide scanner, whole section images were obtained. Morphometric measurements of pulmonary small arteries were performed using NDP viewer (Hamamatsu) in ten random selected digital sections of 2 × 1 mm per animal. Vessels (10–30 μm) were selected and divided in four quadrants, and a quadrant was scored positive for the presence of SMA if more than half of the quadrant was covered [total vessel score ranging from 0 (no SMA) to 4 (completely surrounded by SMA)] by a blinded observer and checked by a second blinded observer.

Endothelin B (ET_B_) receptor staining of the vascular endothelium was performed on 4 µm thick cryo-preserved lung tissue sections, (1:1000 rabbit anti-ET_B_, ab117529, Abcam, Cambridge, UK). Arterioles ranging between 20 and 60 µm in diameter were selected and the lumen divided in four quadrants. Scores were given ranging from 0 (no ET_B_ receptor staining in the endothelium) to 4 (endothelium completely stained for ET_B_ receptor), by a blinded observer and checked by a second blinded observer. If more than half of the quadrant showed endothelial positive staining for the ET_B_ receptor, the quadrant was scored positive.

Pulmonary vascular airway inflammation was assessed using a hematoxylin–eosin (HE) staining on pressure fixated lung tissue in a semi-quantitative way by an experienced, blinded observer. If infiltrates were centered around pulmonary arteries and veins, the inflammation of the areas were scored from 0 to 4, 0—no inflammation; 1—partial diffuse inflammation; 2—generalized diffuse infiltration; 3—diffuse and focal dense infiltration and 4—strong focal dense infiltration. An area of at least 0.5 cm^2^ was evaluated in each slide including bronchial tissue and pulmonary arteries and veins and parenchyma.

### Molecular analyses

Gene expression of several anti-oxidant enzymes, the endothelin system, endothelial and inducible NO synthase, and PDE5 was measured in snapfrozen lung tissue. Total RNA was isolated from bulk lung tissue samples. RNA purity and concentration were measured and cDNA synthesis (SensiFAST cDNA synthesis kit, Bioline, London, UK) was performed using 500 ng RNA as input. Gene expression was analyzed on the CFX96 Real-Time PCR detection system (Biorad, Hercules, CA, USA) using the SensiMix SYBR-green supermix (Bioline). The genes investigated and the primers used are shown in Table 2. Results were normalized to the housekeeping genes RPL13A and Cyclophilin A and relative changes in expression levels were calculated using the BioRad CFX software.

**Table 2:** Genes and primer sequences used for qPCR

Gene	Forward sequence	Backward sequence
CAT	TGCCACCGGCAACTATCCCT	TCGCTGTGAGGCCAAACCTTG
CYPA	AGACAGCAGAAAACTTCCGTG	AAGATGCCAGGACCCGTATG
ECE-1	CTGCAGGCACCGTTCTACACC	CCACGACGACGCCGATGCCAC
EDNRA	TCTGCGCTCTCAGTGTTGAC	AGCCGATTGCTTCAGGGATG
EDNRB	GGAAATCGCCTGCGAATCTG	TGGCTAGTGGCAAGCAGAAA
GPX1	ACCGACCCCAAGTTTATCAC	CATCAGGTGTTCCTCCACA
NOS2	TCCAGGCAATGGAGAGAAAC	CCGAACACAGCATACCTGAA
NOS3	GGACACACGGCTAGAAGAGC	TCCGTTTGGGGCTGAAGATG
PDE5	GCCACTCAATCATGGAGCATC	GGAGAGGCCACTGAGAATCTG
PPET	TTCATCGGCAGCTGGTGATGG	CTTATCTCTGTAGAGCTCGGC
RPL13A	TGGCCAAGCAGGTACTTCTG	GTATTCATGCGCTTGCGGAG
SOD1	CATTCCATCATTGGCCGCAC	CCCAATTACACCACAGGCCA
SOD2	GGCCTACGTGAACAACCTGA	TGATTGATGTGGCCTCCACC
SOD3	CAGACACACTCTCCGCTTCT	AGACCTTCGGGGTAAATGG

*CAT* catalase, *CYPA* cyclophilin A, *ECE-1* endothelin converting enzyme, *EDNRA* endothelin receptor A, *EDNRB* endothelin receptor B, *GPX1* glutathione peroxidase, *NOS2* inducible nitric oxide synthase, *NOS3* endothelial nitric oxide synthase, *PDE5* phosphodiesterase 5, *PPET* prepro-endothelin 1, *RPL13A* ribosomal protein L13a, *SOD* superoxide dismutase

Total endothelial NOS (eNOS), phosphorylated eNOS (Ser1177 site), vasodilator-stimulated phosphoprotein (VASP), phosphorylated VASP, eNOS monomer and dimer protein levels were determined in frozen, homogenized bulk pulmonary tissue samples. For detection of eNOS monomer and dimer fractions, low temperature SDS–PAGE in the absence of β-mercaptoethanol was performed as previously described [50]. Briefly, gels and buffers were equilibrated at 4 °C before electrophoresis, and the buffer tank was placed in an ice bath during electrophoresis to maintain the low temperature. SDS–PAGE for phosphorylated eNOS, total eNOS protein content and housekeeping protein GAPDH was performed at room temperature. Following SDS–PAGE, the proteins were transferred to nitrocellulose membranes and the blots were probed with primary anti-phospho eNOS Ser1177 (1:1000, purified monoclonal rabbit anti-human eNOS, CST9570, Cell Signalling Technology Inc., Danvers, MA, USA), anti-eNOS (1:500, purified monoclonal Mouse anti-human eNOS, 610297, Transduction Laboratory, BD Biosciences, San Jose, CA, USA), anti-VASP (1:1000, purified monoclonal rabbit anti-human VASP, CST3132, Cell Signalling Technology Inc.) anti-phospho VASP Ser239 (1:1000, purified polyclonal rabbit anti-human VASP, CST3114, Cell Signalling Technology Inc.) and anti-GAPDH (1:1000, 14C10, Cell Signalling Technology Inc.). All blots were analyzed using the Odyssey CLX imaging system (LI-COR Biotechnology, Lincoln, NE, USA).

### Data analysis and statistics

Off-line analysis of hemodynamics was performed using CODAS and Matlab. Hemodynamic data were averaged over 10 s. Body O_2_ consumption indexed for body weight (BVO_2_) was computed by (cardiac output/body weight) × (arterial O_2_ content − mixed venous O_2_ content). SVR was calculated as mean arterial pressure/(cardiac output/body weight). Transpulmonary gradient (TPG) was computed by mean pulmonary arterial pressure − mean left atrial pressure. Pulmonary vascular resistance indexed for body weight (PVR) was computed by (mean pulmonary arterial pressure − left atrial pressure)/(cardiac output/body weight). Ea was computed by mean pulmonary arterial pressure/(stroke volume/body weight). Compliance was computed by (stroke volume/body weight)/(systolic − diastolic pulmonary pressure). Data were tested for normality using the Shapiro–Wilk and Kolmogorov–Smirnov test. Data showing a normal distribution are presented as mean ± SEM, whereas data without normal distribution are shown as median [interquartile range (IQR)]. Statistical analysis of hemodynamic data was performed in SPSS Statistics 21.0 (IBM Corp, Armonk, NY, USA), using a two-way ANCOVA for treatment effects and between group differences, with BVO_2_ as covariate. Comparison of other variables between the two groups was performed by unpaired Student’s *t* test for parametric data or Mann–Whitney *U* test for non-parametric data. Statistical significance was accepted when *P* ≤ 0.05 (two-tailed), and *P* ≤ 0.10 (two-tailed) was accepted as a statistical trend.

## Results

### Model characteristics

As shown in Table 3, metabolic dysfunction in DM + HC + CKD swine was evidenced by markedly elevated levels of plasma glucose, total cholesterol, LDL/HDL ratio and triglycerides as compared to Healthy swine. Renal dysfunction was present in DM + HC + CKD swine reflected by increased creatinine plasma levels and a significantly lower glomerular filtration rate (GFR). Metabolic and renal dysfunction resulted in a systemic pro-inflammatory state as indicated by a higher TNF-α and ET-1 plasma levels.

**Table 3:** Metabolic, renal, inflammatory parameters and pulmonary anti-oxidant systems of Healthy and DM + HC + CKD swine

	Healthy (*n* = 18)	DM + HC + CKD (*n* = 19)
Body weight (kg)	98 ± 3	94 ± 4
Metabolic function		
Plasma fasting glucose (mmol L^−1^)	7.2 ± 0.6	21.3 ± 1.1*
Plasma total cholesterol (mmol L^−1^)	1.80 (1.60–2.25)	11.80 (7.30–21.20)*
LDL/HDL cholesterol ratio	1.22 (1.04–1.37)	2.97 (1.81–4.43)*
Plasma triglycerides (mmol L^−1^)	0.23 (0.16–0.28)	0.47 (0.27–0.82)*
Renal function		
Plasma creatinine (µmol L^−1^)	126 (107–135)	157 (136–179)*
GFR (ml min^−1^)^a^	196 ± 11	129 ± 12*
Circulating (anti-) inflammatory factors		
TNF-α (pg mL^−1^)^b^	29 (22–56)	65 (56–116)*
Endothelin 1 (pg mL^−1^)^c^	27 ± 2	33 ± 2*
Nitrite + nitrate (µmol L^−1^)^d^	3.33 ± 0.52	3.78 ± 0.89
Lung NO system^e^		
eNOS	0.0027 ± 0.0006	0.0023 ± 0.0003
iNOS	0.0012 (0.0010–0.0025)	0.0019 (0.0003–0.0025)
PDE5	0.093 (0.072–0.106)	0.122 (0.075–0.173)
Lung endothelin system^e^		
PPET	0.020 ± 0.004	0.032 ± 0.011
ECE-1	0.023 ± 0.001	0.028 ± 0.003
EDNRA	0.017 (0.013–0.012)	0.017 (0.012–0.023)
EDNRB	0.048 ± 0.008	0.039 ± 0.007
EDNR A/B ratio	0.40 (0.32–4.2)	0.39 (0.30–0.75)
Lung antioxidants^e^		
Catalase	0.132 ± 0.011	0.185 ± 0.018*
SOD1	0.340 (0.288–0.538)	0.129 (0.125–0.167)*
SOD2	0.081 (0.071–0.135)	0.066 (0.052–0.155)
SOD3	0.063 ± 0.013	0.039 ± 0.011
Glutathione peroxidase	0.463 ± 0.085	0.792 ± 0.133^(^*^)^

Values are mean ± SEM or median (IQR)

*GFR* glomerular filtration rate, *TNF-α* tumor necrosis factor alpha, *eNOS* endothelial nitric oxide synthase, *iNOS* inducible nitric oxide synthase, *PDE5* phosphodiesterase 5, *PPET* prepro-endothelin 1, *ECE-1* endothelin converting enzyme, *EDNRA* endothelin receptor A, *EDNRB* endothelin receptor B, *SOD* superoxide dismutase

*P ≤ 0.05, ^(^*^)^*P* ≤ 0.1 Healthy vs DM + HC + CKD

^a^Healthy *n* = 9 DM + HC + CKD *n* = 9

^b^Healthy *n* = 14 DM + HC + CKD *n* = 17

^c^Healthy *n* = 14 DM + HC + CKD *n* = 15

^d^Healthy *n* = 13 DM + HC + CKD *n* = 11

^e^Healthy *n* = 6 DM + HC + CKD *n* = 5

### Pulmonary and systemic hemodynamics

**Fig. 1:**
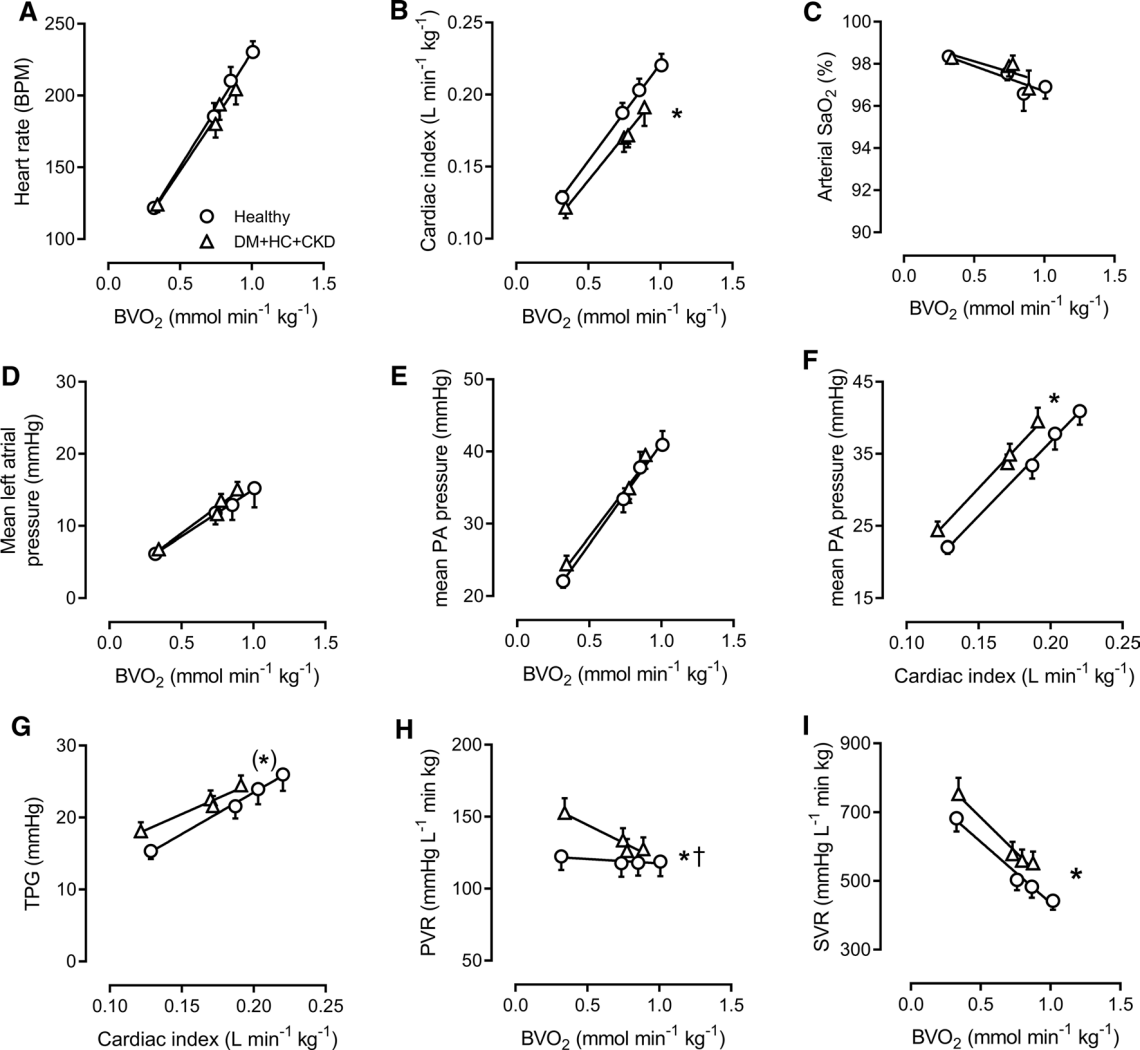
Pulmonary hemodynamic and function. Heart rates at rest and during exercise were similar between groups (**A**), cardiac index was lower in DM + HC + CKD compared to Healthy swine (**B**) for any given level of body oxygen consumption (BVO_2_). Pulmonary function was maintained as oxygen saturation (SaO_2_, **C**) was similar between groups. Left atrial pressures were similar between groups, suggesting no left ventricular backward failure (**D**). Mean pulmonary arterial (PA) pressure was similar for any given level of BVO_2_ (**E**), but was increased for any given level of filling (**F**). Transpulmonary gradient (TPG) was also increased for any level of cardiac index (**G**), resulting in a higher pulmonary vascular resistance (PVR) in DM + HC + CKD compared to Healthy (**H**). Systemic vascular resistance (SVR) was higher in DM + HC + CKD compared to Healthy (**I**). PVRi *n* = 11, rest of parameters *n* = 12 for both groups. Values are mean ± SEM. *P ≤ 0.05, ^(^*^)^*P* ≤ 0.1 for Healthy versus DM + HC + CKD, ^†^*P* ≤ 0.05 for interaction DM + HC + CKD × BVO_2_

**Fig. 2:**
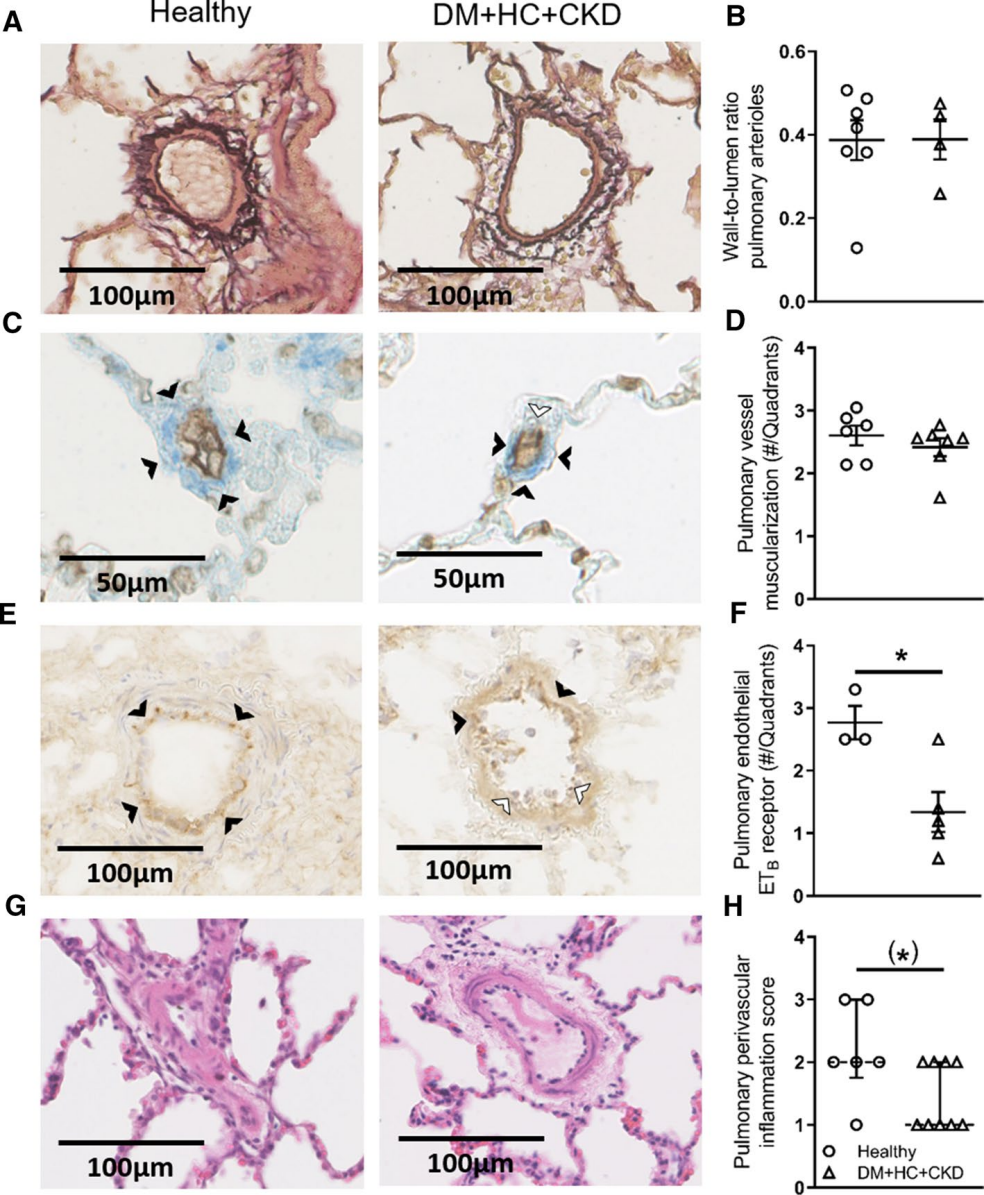
Pulmonary vessel structure of DM + HC + CKD and Healthy swine. Typical examples of pulmonary elastin stained (**A**), lectin and smooth muscle stained (**C**), Endothelin B (ET_B_) receptor stained (**E**) and hematoxylin–eosin stained (**G**) sections. Wall-to-lumen ratio of pulmonary arterioles (**B**) and muscularization of small (10–30 µm) pulmonary vessels (0—non-muscularized; 1, 2, 3—partially muscularized and 4—fully muscularized) (**D**) was not different between groups. Number of quadrants stained for the endothelin B (ET_B_) receptor in the pulmonary endothelium of arterioles (20–60 µm) were lower (**F**) and perivascular inflammation score tended to be lower (**H**) in DM + HC + CKD compared to Healthy (**H**). Black arrows show scored quadrants in panel **C** and **E**, white arrows represent unscored quadrants. Values are mean ± SEM (solid line for panel **B**, **D** and **F**), or median ± IQR (dashed line for panel **H**). *P ≤ 0.05, ^(^*^)^*P* ≤ 0.10 for Healthy versus DM + HC + CKD

Although the exercise-induced increase in heart rate was blunted in DM + HC + CKD swine, the relation between heart rate and body oxygen consumption (BVO_2_) was unaltered compared to Healthy swine (Fig. 1). Mean arterial pressure (at rest: 88 ± 3 mmHg in Healthy vs 87 ± 2 mmHg in DM + HC + CKD) and left atrial pressure were also similar, but cardiac index was lower for any given level of BVO_2_, indicating an increased systemic vascular resistance (SVR) (Fig. 1). Respiratory function was maintained as arterial oxygenation was similar between groups both at rest and during exercise, (Fig. 1). Furthermore, although the relation between mean PA pressure and BVO_2_ was similar in DM + HC + CKD swine compared to Healthy swine, the relations between PA pressure and cardiac index, as well as the relation between TPG and cardiac index were shifted upwards, reflecting a higher pulmonary vascular resistance (PVR) in DM + HC + CKD swine, particularly under resting conditions (Fig. 1). This increase in PVR could not be explained by structural pulmonary arterial changes, as pulmonary arterial compliance (38 ± 2 vs. 37 ± 3 μl∙kg^−1^∙mmHg^−1^ in Healthy and DM + HC + CKD, respectively) and media-to-lumen ratio in vessels of ~ 80 µm diameter as well as muscularization of vessels ranging 10–30 µm in diameter were similar between Healthy and DM + HC + CKD (Fig. 2), suggesting that the increase in PVR was mediated by functional alterations, i.e. an increase in pulmonary vascular tone.

### Alterations in pulmonary and systemic vascular tone regulation

**Fig. 3:**
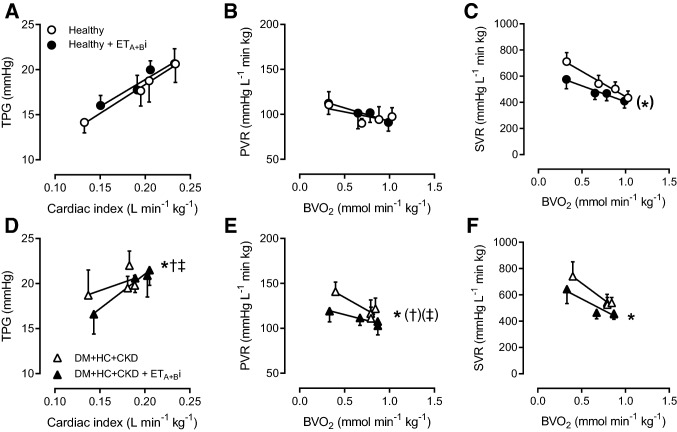
Role of the endothelin-pathway in pulmonary and systemic vasomotor control in DM + HC + CKD and Healthy swine. Transpulmonary gradient (TPG) as a function of cardiac index during graded exercise showed no significant change in response to endothelin receptor A and B blockade (ET_A + B_i) in Healthy (A) but did decrease slightly in DM + HC + CKD (D) swine particularly at rest. Pulmonary vascular resistance (PVR) was unchanged in response to ET_A + B_i in Healthy (**B**), while DM + HC + CKD showed pulmonary vasodilation in response to ET_A + B_i (**E**). ET_A + B_i resulted in similar reduction in systemic vascular resistance (SVR) in Healthy and DM + HC + CKD (**C**, **F**). Healthy *n* = 6, DM + HC + CKD *n* = 6. Values are mean ± SEM. *P ≤ 0.05, ^(*)^*P* ≤ 0.1 for effect ET_A + B_i within group, ^†^*P* ≤ 0.05, ^(†)^*P* ≤ 0.10 for interaction ET_A+B_i × cardiac index (**C**) or ET_A + B_i × BVO_2_ (**D**) within groups, ^‡^*P* ≤ 0.05, ^(‡)^*P* ≤ 0.10 for interaction ET_A+B_i × group

Despite elevated circulating ET-1 levels, ET_A + B_i induced similar vasodilation of the systemic vasculature in DM + HC + CKD and Healthy swine (Fig. 3). ET_A + B_i with tezosentan had no effect on PVR in Healthy swine (Fig. 3). In contrast, ET_A + B_i resulted in pulmonary vasodilation in DM + HC + CKD swine, as evidenced by a significant decrease in PVR and TPG (Fig. 3). Interestingly, in the presence of ET_A + B_i, the TPG and PVR values in DM + HC + CKD were similar to the control values and the ET_A + B_i values in the Healthy group. These observations indicate that an increased pulmonary vasoconstrictor influence of ET was principally responsible for the increased PVR in DM + HC + CKD. Although the mRNA levels of the ET_A_ and ET_B_ receptors, prepro-ET-1 and endothelin converting enzyme were unaltered in bulk lung tissue (Table 3), immunohistochemical staining for the ET_B_ receptor in a subset of animals showed a reduced ET_B_ expression in the pulmonary endothelial cells of DM + HC + CKD swine (Fig. 2). Since the endothelial ET_B_ receptor is the main clearance receptor for ET, and its activation induces NO-mediated vasodilation, such reduced ET_B_ expression likely contributed to the enhanced circulating ET-levels and ET-mediated pulmonary vasoconstrictor influence.

**Fig. 4:**
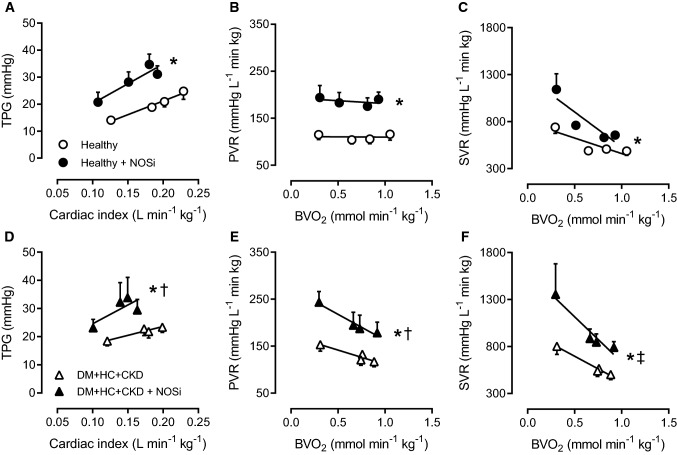
Nitric oxide in pulmonary and systemic vasomotor control in DM + HC + CKD and Healthy swine. Transpulmonary gradient (TPG) as a function of cardiac index markedly increased as a result of endothelial nitric oxide synthase inhibition (NOSi) in both Healthy (**A**) and DM + HC + CKD (**D**) both at rest and during exercise. NOSi resulted in a similar increase in pulmonary vasoconstriction, as evidenced by the increase in pulmonary vascular resistance (PVR), in Healthy (**B**) and DM + HC + CKD swine (**E**). Nitric oxide synthase inhibition (NOSi) has a more pronounced effect on systemic vascular resistance (SVR) in DM + HC + CKD (**F**) than in Healthy swine (**C**). Healthy *n* = 7, DM + HC + CKD *n* = 7 at rest and *n* = 5 during exercise. Values are mean ± SEM. *P ≤ 0.05 for effect NOSi within group, ^†^*P* ≤ 0.05 for interaction NOSi × cardiac index (**D**) or NOSi × BVO_2_ (**E**) within group. ^‡^*P* ≤ 0.05 for interaction NOSi × group (**F**)

**Fig. 5:**
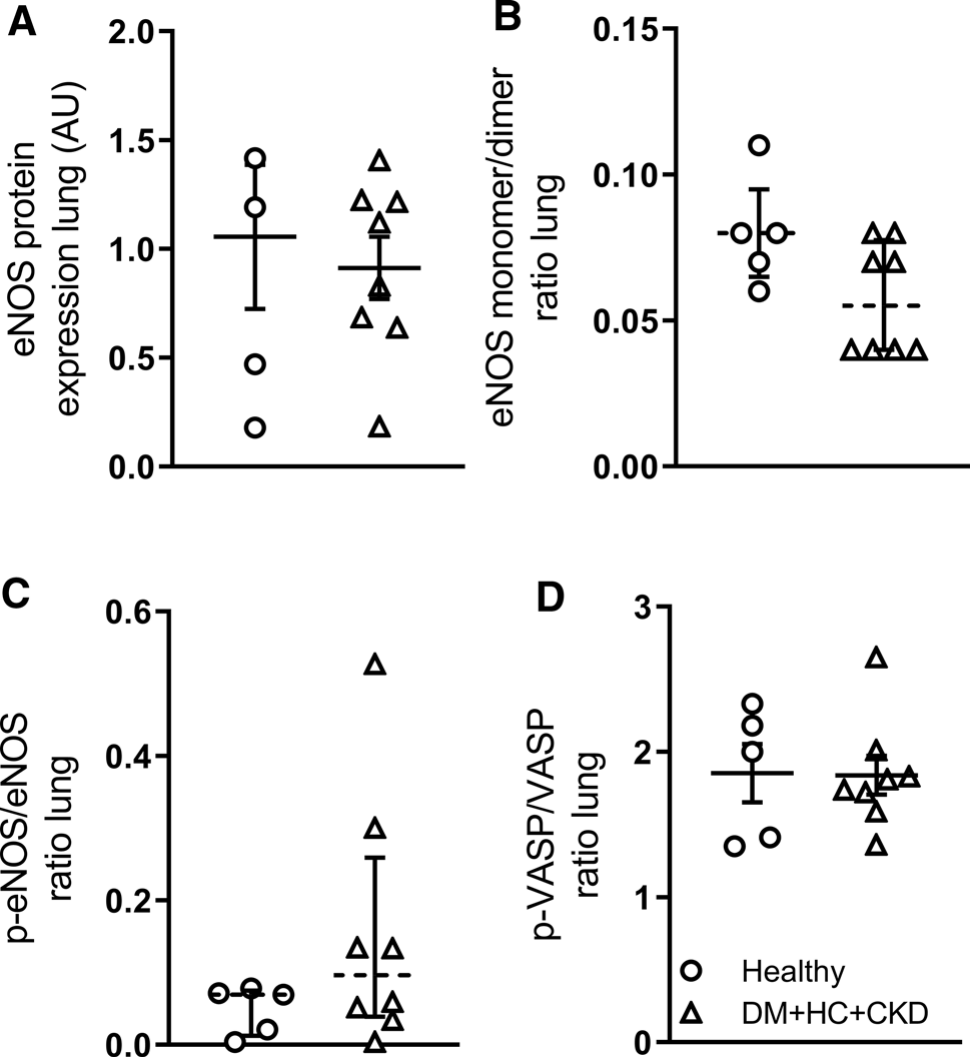
Endothelial nitric oxide (eNOS) pathway activity patterns in bulk lung tissue of DM + HC + CKD and Healthy, detected by Western blots. Total eNOS protein (**A**), eNOS monomer/dimer ratios (**B**), phosphorylated eNOS/eNOS ratios (**C**) and phosphorylated VASP/VASP ratios (**D**) were unchanged in DM + HC + CKD compared to Healthy. Values are mean ± SEM (solid lines, panel **A** and **D**) or median ± IQR (dashed lines, panel **B** and **C**). Uncut Western blots are provided in the supplemental data file

**Fig. 6:**
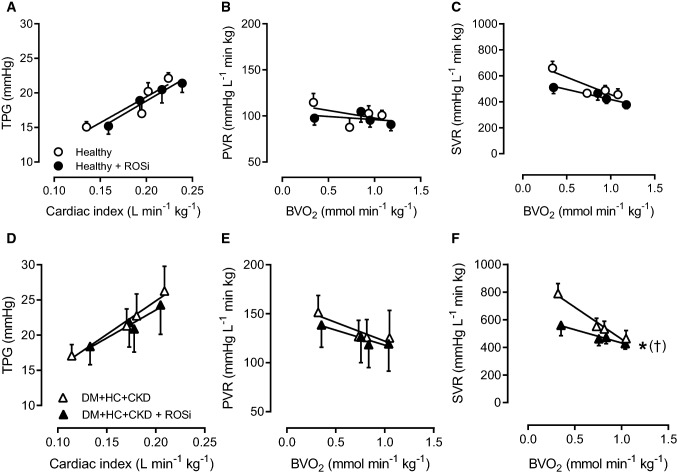
Role of scavenging of reactive oxygen species (ROSi) in pulmonary and systemic vasomotor control in DM + HC + CKD and Healthy swine. Transpulmonary gradient (TPG) as a function of cardiac index did not result in significant change after ROSi in both Healthy (**A**) and in DM + HC + CKD (**D**). Pulmonary vascular resistance (PVR) showed no response to ROSi in Healthy (**B**), and in DM + HC + CKD (**E**). In the systemic vasculature, no effect of ROSi was observed in Healthy **(C)**, but resulted in vasodilatation in DM + HC + CKD **(F)** at rest, while this effect was lost during exercise. Healthy *n* = 8, DM + HC + CKD *n* = 8. Values are mean ± SEM. *P ≤ 0.05 for effect ROSi within group, ^(†)^*P* ≤ 0.10 for interaction ROSi × BVO_2_ within group

NOSi markedly increased TPG while decreasing cardiac output at rest, reflecting an increase in PVR, this effect being similar in Healthy and DM + HC + CKD swine (Fig. 4). The increase in PVR induced by NOSi was slightly attenuated during exercise in DM + HC + CKD but not Healthy swine (Fig. 4). The maintained vasodilator influence of endogenous NO was consistent with the unaltered eNOS protein levels, eNOS uncoupling (monomer/ dimer ratio) and eNOS phosphorylation as well as with the unaltered phosphorylated VASP/VASP ratio in lung tissue, which reflects similar PKG activity between groups (Fig. 5). Furthermore, iNOS mRNA was unchanged and approximately threefold lower than eNOS mRNA levels (Table 3). However, several important anti-oxidant systems were affected by DM + HC + CKD, with increased catalase and glutathione peroxidase mRNA expression, but decreased superoxide dismutase 1 (SOD1) expression in the lungs of DM + HC + CKD swine (Table 3). This was accompanied by a trend towards a decrease in pulmonary perivascular inflammation score (Fig. 2). ROS scavenging with a combination of MPG and Tempol did not result in changes in PVR in either Healthy or DM + HC + CKD swine at rest or during exercise (Fig. 6), suggesting that ROS do not influence pulmonary vascular tone in this model. Conversely, ROS scavenging did induce systemic vasodilation in DM + HC + CKD but not Healthy swine (Fig. 6). Furthermore, NOSi induced more vasoconstriction in the systemic circulation in DM + HC + CKD compared to Healthy swine (Fig. 4).

**Fig. 7:**
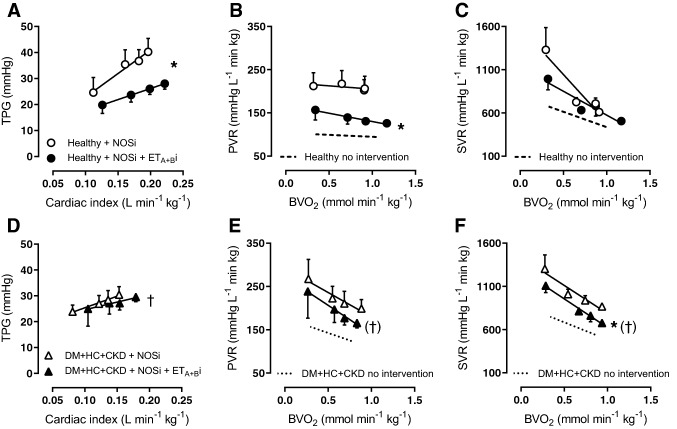
Role of Endothelin 1 after eNOS inhibition in pulmonary and systemic vasomotor control in DM + HC + CKD and Healthy swine. Transpulmonary gradient (TPG) as a function of cardiac index decreased significantly after endothelin receptor A and B inhibition (ET_A + B_i) in the presence of NOS inhibition (NOSi) in Healthy (**A**) but not in DM + HC + CKD (**D**). Pulmonary vascular resistance (PVR) decreased significantly in response to ET_A + B_i after NOSi in Healthy (**B**), while in DM + HC + CKD (**E**) ET_A + B_i additional to NOSi did not result in pulmonary vasodilation. In the systemic vasculature, effect of ET_A + B_i during NOSi was only observed at rest in Healthy **(C)**, but resulted in vasodilatation at rest and during exercise in DM + HC + CKD **(F)**. Healthy *n* = 6, DM + HC + CKD *n* = 4. Values are mean ± SEM. *P ≤ 0.05 for effect ET_A + B_i within group, ^†^*P* ≤ 0.05, (^†^)*P* ≤ 0.10 for interaction ET_A+B_i × group

The NO and the ET-pathways interact with each other at multiple levels, i.e. activation of ET_B_ receptors on endothelial cells results in NO production and NO suppresses ET production and release, both resulting in NO interfering with ET-mediated vasoconstriction. Hence, ET_A + B_i was repeated in the presence of NOSi. Following NOSi, ET_A + B_i decreased SVR in DM + HC + CKD but not Healthy swine, which suggests that, indeed, increased ET-mediated systemic vasoconstriction contributed to the larger increase in SVR in response to NOSi in DM + HC + CKD (Fig. 7).

In the pulmonary vasculature of Healthy swine, NOSi unmasked a vasodilator effect of ET_A + B_i, as evidenced by marked reductions in TPG and PVR at rest and during exercise (Fig. 7). In swine with DM + HC + CKD, ET_A + B_i did not result in a statistically significant change in PVR in the presence of NOSi (Fig. 7). Together with the reduced endothelial ET_B_ receptor expression in the lung of DM + HC + CKD (Fig. 2), these data imply that in Healthy swine, the ET_B_ receptor activates NO production, thereby suppressing the pulmonary vasoconstrictor influence of ET, and that this effect is lost in DM + HC + CKD swine.

**Fig. 8:**
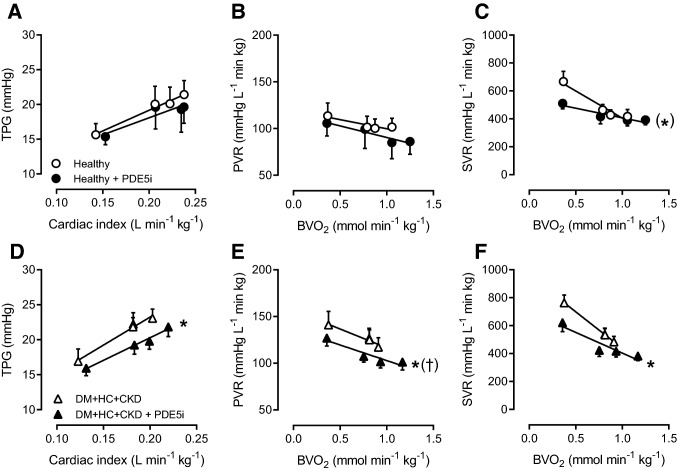
Phosphodiesterase 5 (PDE5) in pulmonary and systemic vasomotor control in DM + HC + CKD and Healthy swine. The relation between transpulmonary gradient (TPG) and cardiac index shifted downward as a result of PDE5 inhibition (PDE5i) in DM + HC + CKD (**C**) but not in Healthy (**A**). PDE5i resulted in pulmonary vasodilation in DM + HC + CKD (**D**) but not in Healthy swine (**B**) as evidenced by the decrease in pulmonary vascular resistance (PVR). The reduction in systemic vascular resistance (SVR) with administration of PDE5i was similar between both groups. Healthy *n* = 6, DM + HC + CKD *n* = 7. Values are mean ± SEM. *P ≤ 0.05, ^(^*^)^*P* ≤ 0.1 for effect PDE5i within group, (^†^)*P* ≤ 0.1 for interaction PDE5i*group

Further downstream in the NO-pathway, inhibition of PDE5 acts to prolong the half-life of cGMP. Despite an unaltered PDE5 mRNA expression in lung tissue (Table 3), PDE5i decreased TPG and PVR in DM + HC + CKD swine but not in Healthy swine (Fig. 8), both at rest and during exercise. The increased vasoconstrictor influence of PDE5 in the pulmonary vasculature likely also contributed to the increased PVR in DM + HC + CKD swine. In contrast, the reduction in SVR with PDE5i was similar between groups (Fig. 8).

### Right ventricular function and structure

**Fig. 9:**
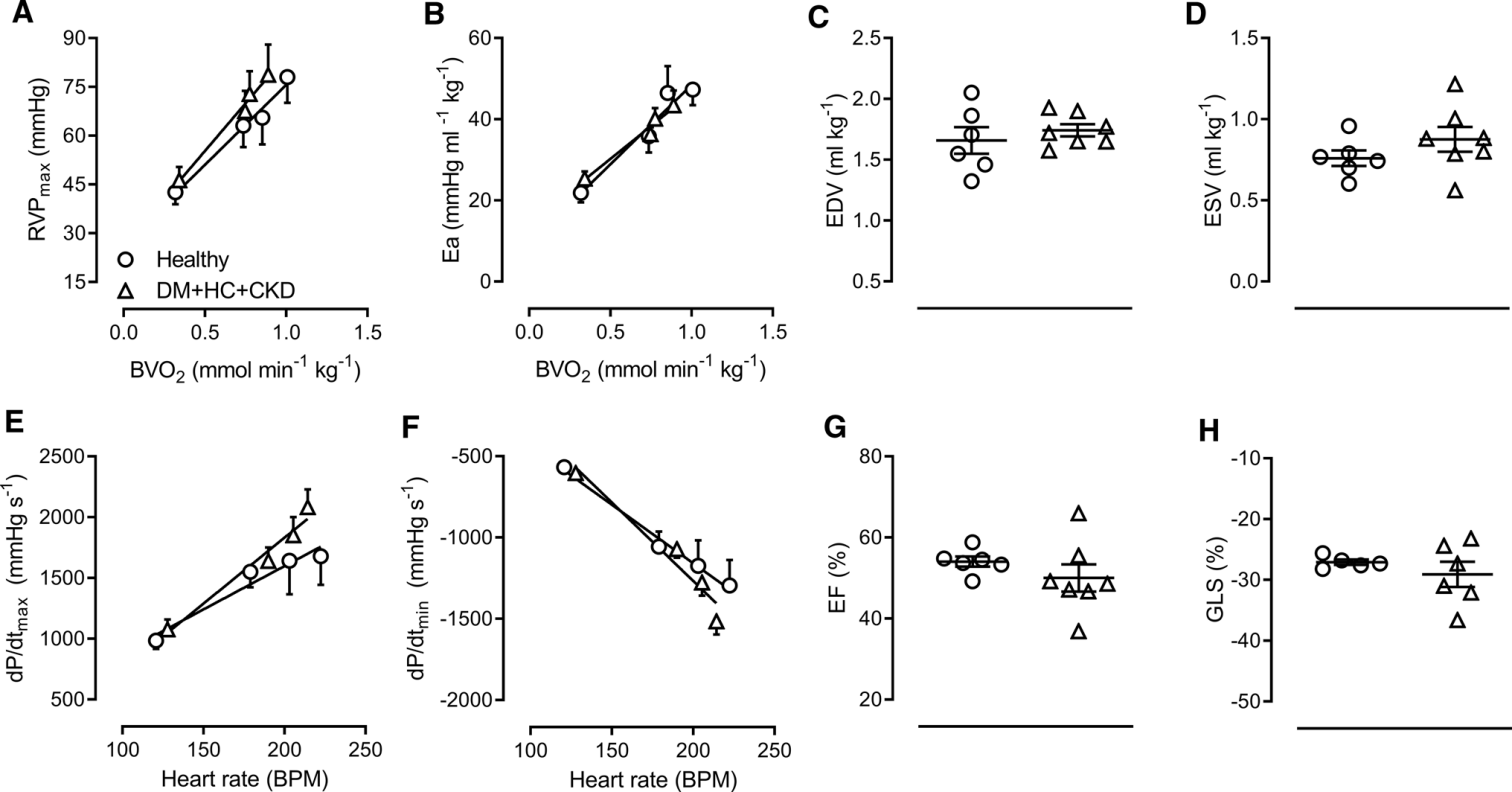
Right ventricular function at rest, during exercise and during cardiovascular magnetic resonance imaging in DM + HC + CKD and Healthy swine. Right ventricular afterload at rest and during exercise was similar as the maximal right ventricular pressure (RVP_max_, **A**) and pulmonary arterial elastance (Ea, **B**) were similar between groups for any given level of body oxygen consumption (BVO_2_). Systolic (d*P*/d*t*_max_; maximum of the first derivate of the systolic RV pressure increase, **E**) and diastolic function (d*P*/d*t*_min_; minimum of first derivate of the RV diastolic pressure decrease, **F**) were preserved in DM + HC + CKD swine for any level of heart rate. *n* = 11 for Healthy and *n* = 10 for DM + HC + CKD. Right ventricle cardiovascular magnetic resonance imaging parameters were measured under general anesthesia. End-diastolic (EDV, **C**) and end-systolic volume (ESV, **D**) ejection fraction (EF, **G**) and global longitudinal strain (GLS, **H**) were unaltered in DM + HC + CKD (*n* = 7) compared to Healthy swine (*n* = 6). Values are mean ± SEM

In accordance with the unaltered PA pressure, pulmonary vascular elastance (Ea), a measure of RV afterload was not changed in DM + HC + CKD (Fig. 9). RV systolic and diastolic functions were maintained in DM + HC + CKD and RV diastolic and systolic volumes were also unaltered (Fig. 9).

**Fig. 10:**
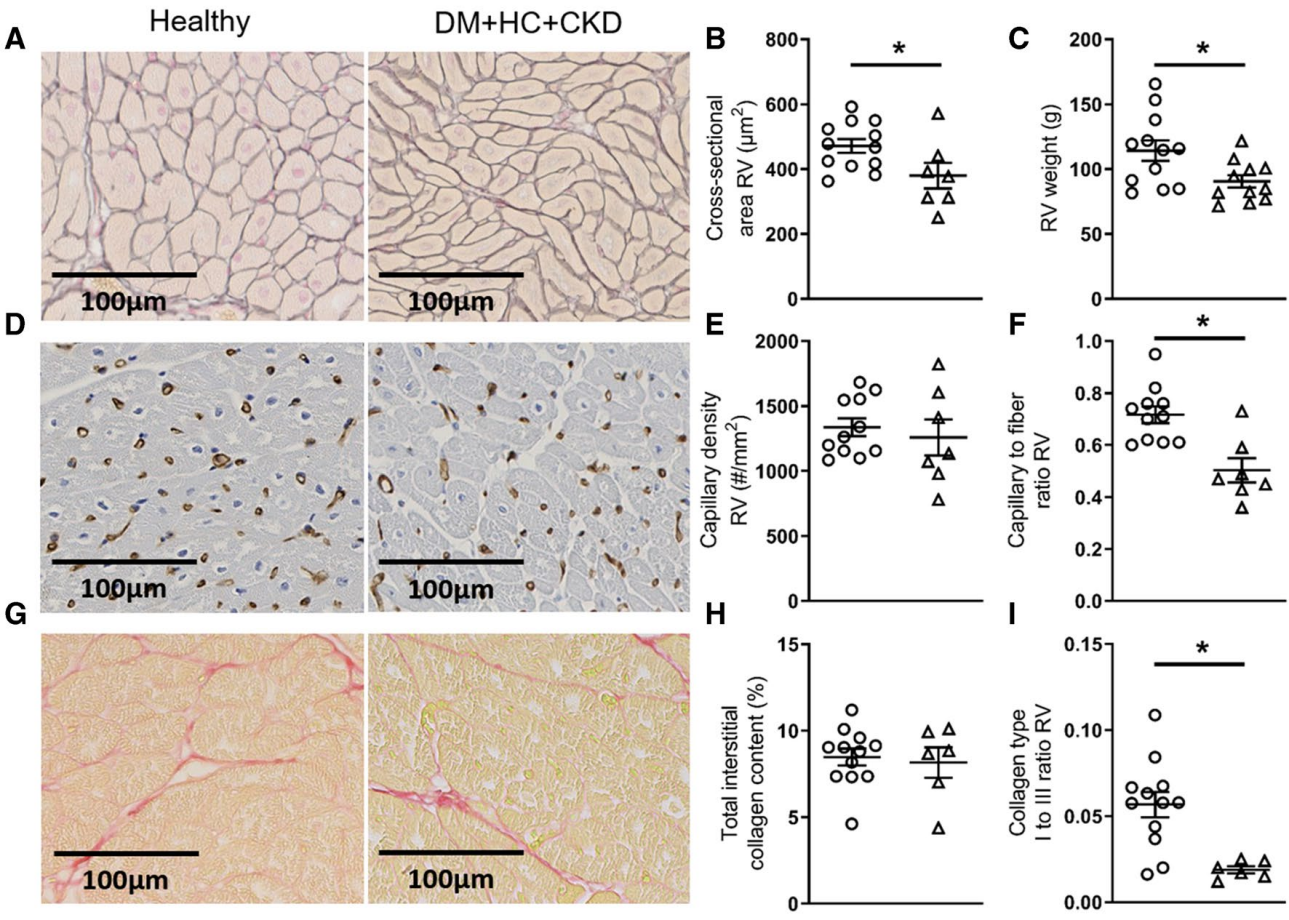
Right ventricular structure of DM + HC + CKD and Healthy swine typical examples of right ventricular Gomori stained (**A**), Lectin stained (**D**) and Picrosirius red stained (**G**) sections. The cross-sectional area of the RV cardiomyocytes was decreased in DM + HC + CKD swine (**B**), RV weight was also lower in DM + HC + CKD swine (**C**). Capillary density was similar between groups (**E**) but capillary-to-fiber ratio was lower in DM + HC + CKD (**F**). Total interstitial collagen content (**H**) was unaltered in DM + HC + CKD swine, but there was a shift the composition of the specific collagen fibers in DM + HC + CKD (**I**). Values are mean ± SEM. *P ≤ 0.05 for Healthy versus DM + HC + CKD, scale bars are 100 µm

Histological examination of the RV revealed subtle changes in the structure of the RV. Interestingly, the cross-sectional area of the cardiomyocytes was decreased in DM + HC + CKD compared to Healthy swine (Fig. 10). Absolute RV weights were also lower in DM + HC + CKD than Healthy swine (Fig. 10), while relative RV weight to total heart weight (Healthy 0.29 ± 0.01 vs DM + HC + CKD 0.27 ± 0.01, *P* = 0.196) and the Fulton index (Healthy 0.42 ± 0.02 vs DM + HC + CKD 0.37 ± 0.02, *P* = 0.177) were unchanged. Additionally, capillary density was similar in both groups (Fig. 10). In line with these findings, combination with the cell count of the cardiomyocytes revealed a decreased capillary-to-fiber ratio in DM + HC + CKD compared to Healthy controls (Fig. 10). The total collagen content of the RV was similar between the groups, however, a shift of the collagen type composition of the RV towards more compliant type III collagen was observed (Fig. 10).

## Discussion

The present study tested the hypothesis that in a swine model with multiple comorbidities and left ventricular diastolic dysfunction, in the absence of overt left ventricular backward failure, multiple comorbidities result in functional pulmonary vascular alterations in exercising swine. The main findings were that (i) the combination of DM, HC and CKD resulted in increased systemic and pulmonary vascular resistance. (ii) The increase in pulmonary vascular resistance was principally due to changes in pulmonary vasomotor control in the absence of pulmonary vascular structural changes. (iii) ET_A+B_i as well as PDE5i reduced PVR in DM + HC + CKD but not in Healthy swine. (iv) The increase in PVR in response to NOSi was similar in DM + HC + CKD compared to Healthy swine, with unaltered eNOS protein, phosphorylation and uncoupling. Furthermore, eNOSi unmasked a vasodilator effect of subsequent ET_A + B_i in Healthy swine, but not in DM + HC + CKD swine, consistent with the observed loss of pulmonary endothelial ET_B_ receptors. (v) ROS scavenging did not induce pulmonary vasodilation in either DM + HC + CKD or Healthy swine. The implications of these findings are discussed below with a focus on PVD as an early complication of DM + HC + CKD.

### Methodological considerations

HFpEF is a heterogeneous disorder resulting from various combinations of underlying comorbidities that is more prevalent in women [10, 43]. To resemble the clinical pathogenesis of HFpEF, the current study was performed in female swine with multiple common comorbidities. Swine have been utilized in multiple investigations concerning metabolic derangement as lipid and glucose metabolism resemble that in humans, with comparable triglycerides levels in high-fat diet induced hypercholesterolemia [2]. Additionally, repeated streptozotocin injections induced hyperglycemia without insulin-dependency mimicking a late-type 2 DM with insulin resistance as seen in patients [55]. Renal dysfunction is present in approximately 50% of HFpEF patients and diastolic dysfunction is one of the first observed cardiovascular alterations in patients with early-stage CKD [54]. In the present study, CKD was induced by embolizing ~ 3/4 of the kidneys and resulted in a 30% decrease in renal function mimicking an early stage of CKD. Our findings, which indicate that PVD is already present in swine after 5–6 months of exposure to multiple comorbidities—including CKD—are in accordance with two studies using unsupervised phenomapping of a large group of patients clinically diagnosed with HFpEF [47, 61]. This phenomapping resulted in three or four main phenotypes, in which the presence of CKD clustered with RV dilation and high pulmonary pressures in the group with the worst prognosis [47, 61]. Moreover, the fact that renal dysfunction, pulmonary and peripheral vascular alterations in HFpEF were recently prioritized on the research agenda for the coming decade by the National Heart, Lung, and Blood Institute Working Group on HFpEF [46], underscores the value of animal models to further study their interaction. Our early observations warrant future studies in swine using longer periods of follow-up to investigate whether the PVD already found in the present study at 6 months, progresses to overt pulmonary hypertension and right heart failure.

One aspect of HFpEF that was not accounted for in the present study, is that HFpEF is predominantly present in older post-menopausal women. Indeed, sex-differences and sex-hormones are well-known to play a role in the regulation of vascular tone as well as in the development and progression of cardiovascular disease [45], and differences in the ET system have been proposed to play a role in this sexual dimorphism. Thus, premenopausal women have lower circulating ET-levels as compared to age matched men, but ET-levels increase after menopause, which has been ascribed to estrogen stimulating eNOS, which in turn inhibits the ET system [20]. In addition, the ratio of vasodilator ET_B_ receptors to vasoconstrictor ET_A_ receptors is higher in women as compared to men, and ET_A_-mediated vasoconstriction is less pronounced in the forearm of women than men [20]. Future studies should be performed to investigate whether PVD might be aggravated in older and/or ovariectomized swine with multiple comorbidities, and the role of NO- and ET-systems in such potential aggravation.

### Pulmonary vascular disease: an early complication of HFpEF and CKD

The importance of early detection of PVD in various pathologies is increasingly recognized. In pulmonary arterial hypertension, the threshold for PH has recently been lowered from 25 to 20 mmHg [49], as multiple studies showed a higher mortality in patients exhibiting what has previously been described as borderline PH (20–25 mmHg), as well as in patients with PA pressures in the upper normal range at rest (17–19 mmHg) [3, 11]. In other forms of PH, such as HFpEF-associated PH, the debate is ongoing as to whether or not to shift the threshold of PH, since elevations in PA pressure are not solely due to PVD [31, 49]. Nevertheless, also in the HFpEF population, dyspnea was observed prior to pulmonary venous congestion [32], consistent with a pre-capillary PH-phenotype, and hence with the occurrence of PVD as an initiating factor. One of the earliest signs of PVD in these patients is an exaggerated increase in PA pressure during stress, which is out of proportion with the increases in left atrial pressure and cardiac output, and reflects the inability to decrease PVR during exercise [21, 31].

In our study, the relation between cardiac index and PA pressure was shifted upwards due to an increased TPG, and PVR was higher at all levels of exercise in swine with DM + HC + CKD as compared to Healthy swine at a time that left atrial pressure was not affected, suggesting microvascular alterations in the pulmonary vasculature. Interestingly, our data show that PVD, evidenced by the elevated PVR, is present even before the increase in SVR and the presence of left ventricular diastolic dysfunction resulting in overt elevations in left atrial pressure or PA pressure. However, already at this time point, a more generalized microvascular dysfunction is present as we have recently shown in this porcine model that coronary microvascular function is also impaired [55].

The increased PVR was accompanied by higher circulating levels of TNF-α and ET-1, further reflecting inflammation and endothelial dysfunction. The higher PVR, in the absence of an increase in PA pressure, seems to be in contrast with the general assumption that HFpEF-PH progresses from isolated post-capillary PH due to left ventricular dysfunction, to pre- and post-capillary PH [31, 33]. Although PVD is highly prevalent in HFpEF and increases morbidity and mortality [22, 31], HFpEF patients form a heterogeneous population, with various underlying conditions—such as DM, HC and/or CKD—leading to inflammation and oxidative stress, making some patients more prone to develop combined pre-and post-capillary PH than others. Especially in HFpEF patients suffering from CKD (~ 50% of the HFpEF patients) [52, 54], microvascular dysfunction is partly mediated by overlapping risk factors such as DM and hypertension, but, importantly, uremic toxins can also directly impact the heart and vasculature, making CKD an independent risk factor for HFpEF development [52, 54] as well as for development of PVD and PH [6, 51]. Together with our data these associations suggest that PVD may develop in a subgroup of patients with left ventricular diastolic dysfunction and CKD, prior to and/or independent of increased left atrial pressures. As no overt perivascular inflammation was present in the lungs, and pulmonary vascular muscularization was unaltered, our data suggest that functional and not structural alterations in the pulmonary vasculature are among the first signs of PVD.

### Endothelial dysfunction in early PVD

PVD is characterized by endothelial dysfunction, impaired pulmonary vasomotor control and vascular remodeling. Endothelial dysfunction involves an imbalance between the ET and NO pathways, which may alter pulmonary vascular control. In the current study, the endogenous vasodilator influence of NO was unaltered in the pulmonary vasculature as NOSi resulted in a similar vasoconstrictor response in DM + HC + CKD and Healthy swine. These data are consistent with previous findings from our laboratory, showing that the response to NOSi was maintained in swine with PVD secondary to myocardial infarction [37, 56] and even increased in PVD secondary to pulmonary vein stenosis [58]. In accordance with the maintained vasodilator influence of endogenous NO in the present study, eNOS mRNA, eNOS total protein, uncoupling and phosphorylation were unaltered in DM + HC + CKD. Also, phosphorylated VASP/VASP ratio in lung tissue was unchanged, which reflects similar PKG activity (Fig. 5). Furthermore, circulating nitrite + nitrate levels were similar in both groups (Table 3), although this is a rather insensitive measure that may not reflect the changes in NO bioavailability at the level of the lung vasculature. Similarly, iNOS mRNA, which has been shown to reflect iNOS protein [62], was not increased, and its expression was approximately threefold lower than expression of eNOS mRNA.

Further downstream in the NO-pathway, PDE5i resulted in a pulmonary vasodilation in DM + HC + CKD and normalized PVR, whereas no effect of PDE5i was observed in Healthy swine. The absence of a pulmonary vasodilator effect of PDE5i in Healthy swine is different from previous observations in which we showed a modest pulmonary vasodilator effect of PDE5 inhibition in Healthy swine [56, 58]. This difference is not readily explained, but may be the result of an age difference, as swine in the previous studies were younger than in the present study. Similar to the present study, an increased response to PDE5i has been demonstrated in different swine models of type 2 PH, either due to pulmonary vein stenosis [58] or myocardial infarction [56]. In the present study, we observed no difference in PDE5 mRNA expression, but did not measure PDE5 protein or activity. Increased PDE5 protein expression and activity in the lung vasculature have been observed in experimentally-induced PH in lambs [5]. PDE5 mRNA and protein expression as well as activity in pulmonary vascular smooth muscle cells can be increased by oxidative stress [16, 40]. mRNA expression of the anti-oxidant enzymes catalase and glutathione peroxidase was increased in DM + HC + CKD, but SOD1 was decreased, whereas ROS scavenging with MPG and TEMPOL had no vasoactive effect in the pulmonary vasculature of either Healthy or DM + HC + CKD swine. Nevertheless, upregulation of these anti-oxidant systems may have exerted a protective effect on the pulmonary vasculature, as pulmonary perivascular inflammation tended to be reduced despite higher circulating TNF-α levels and MPG + TEMPOL did exert a vasodilator effect on the systemic vasculature of DM + HC + CKD only. It is known, however, that mRNA levels may not always adequately reflect protein levels. Hence, future studies should confirm these data at the protein level and determine the specific location of the upregulated anti-oxidant enzymes as well as changes in specific oxygen radicals that may have contributed to the increased PDE5 activity.

Conflicting results have been published with respect to the efficacy of PDE5 inhibition in HFpEF-PH, and recent meta-analyses concluded that there was no beneficial effect of PDE5 inhibition on pulmonary hemodynamics or exercise capacity [4, 8], although some individual studies did show a beneficial effect. It should be noted, however, that there were substantial differences in patient populations between studies and that the studies with negative results appeared to have higher proportions of post-capillary PH [24, 26, 44]. Conversely, one study that included patients with a higher PVR, resembling the pre-capillary arterial phenotype observed in this model, showed a beneficial effect of PDE5i [23]. These human data suggest that stratifying patients based on underlying disease may prove useful to identify subgroups of HFpEF-PH patients that may benefit from treatment. Stratification according to the presence of CKD may be necessary as PDE5 inhibition has been shown to either ameliorate [1], or worsen [42] kidney function and may thereby indirectly impact PVD.

Consistent with the increased ET-dependent vasoconstrictor influence on the pulmonary vasculature, circulating ET-1 levels were higher in swine with DM + HC + CKD as compared to Healthy controls suggesting either increased production or impaired clearance. The elevated ET-levels in our swine model are in accordance with human studies, showing elevated plasma ET-levels in HFpEF-PH patients correlating with PA pressures [7, 36, 41, 53], as well as with studies from our laboratory showing that plasma ET-levels are elevated in type II PH in swine, secondary to myocardial infarction [25] or due to pulmonary vein stenosis [58]. Furthermore, wedge ET-1 levels were shown to be higher than pulmonary arterial ET-levels and correlated with PVR in patients with combined pre-and post-capillary but not isolated post-capillary HFpEF-PH [7, 36], suggesting that the higher ET-1 levels in patients with combined pre- and post-capillary PH originate more distally in the pulmonary vasculature.

ET_A_ and ET_B_ receptors are expressed in the pulmonary circulation under both physiological and pathophysiological circumstances [15]. ET_A_ is only present on vascular smooth muscle cells, mediating vasoconstriction, whereas ET_B_ is present on both the vascular smooth muscle cell and the endothelial cells, mediating vasoconstriction and vasodilation, respectively. The lack of effect of ET_A + B_i in Healthy swine may be due to a balance between ET_A_ and/or ET_B_-mediated vasoconstriction and ET_B_-mediated vasodilation [39]. Although we previously showed that young, healthy swine do show a pulmonary vasodilator response to ET_A + B_i [25, 37, 38], this vasodilator response decreased with age [57]. The reason underlying this change is unclear, but may be due to altered ET_A_ and/or ET_B_ receptor expression or function with age. Similarly, in pathophysiological circumstances, ET_A_ and ET_B_ protein expression may be altered and the interaction with the NO-cGMP pathway may be altered [39, 63]. In line with previous observations in swine with type II PH secondary to myocardial infarction [25] and pulmonary vein stenosis [57], ET_A + B_i produced a greater reduction in PVR in swine with DM + HC + CKD, suggesting that the balance between the vasoconstrictor and vasodilator pathways is shifted towards vasoconstriction in the DM + HC + CKD swine, and consistent with a withdrawal of endothelial ET_B_ influence. Indeed, immunohistochemical analysis showed reduced ET_B_ receptor staining on the pulmonary endothelium in DM + HC + CKD swine. Immunohistochemical staining of ET_A_ receptors failed due to lack of a porcine specific antibody suitable for immunohistochemistry. Furthermore, given the partial muscularization of the pulmonary arterioles, with few smooth muscle cells surrounding the lumen, it was not possible to reliably measure ET_B_ receptor staining of the medial layer. Nevertheless, loss of endothelial ET_B_ receptors may result in reduced ET-1 clearance [13, 14], thereby explaining the increased circulating levels of ET-1 found swine with DM + HC + CKD in our study.

**Fig. 11:**
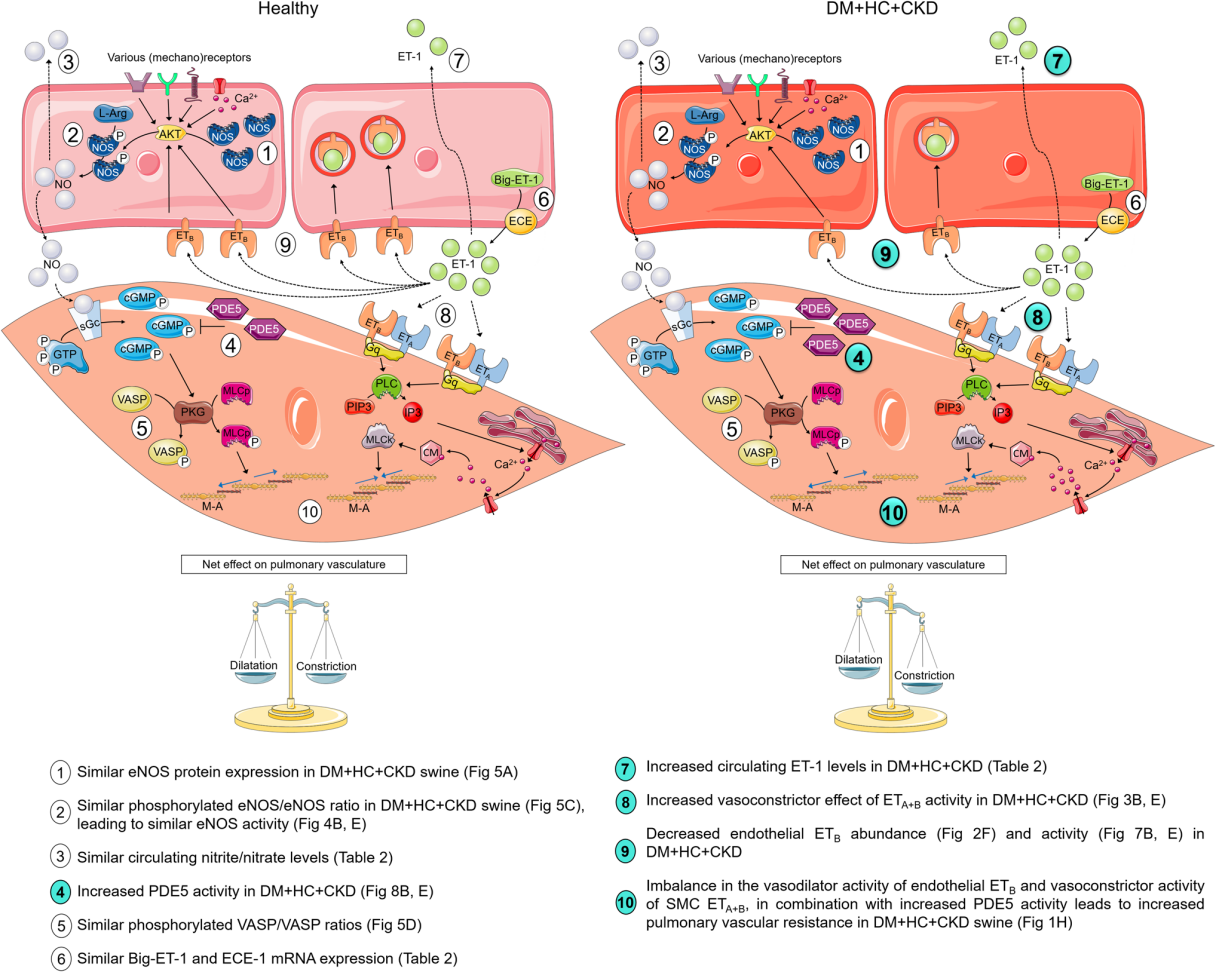
Proposed mechanisms of pulmonary vascular dysfunction in DM + HC + CKD. Upper cells represent endothelial cells, lower cells the smooth muscle cells. Significant differences in DM + HC + CKD compared to Healthy are shown as highlighted numbers in the figure. *AKT* protein kinase B, *Big-ET-1* big endothelin 1, *Ca*^*2+*^ calcium, *cGMP* cyclic guanosine monophosphate, *CM* calmodulin, *ECE* endothelin converting enzyme, *ET-1* endothelin 1, *ET*_*A*_ endothelin receptor A, *ET*_*B*_ endothelin receptor B, *GTP* guanosine triphosphate, *Gq* G alpha subunit, *IP3* inositol triphosphate, *L-Arg* l-arginine, *M-A* myosin–actin, *MLCk* myosin light chain kinase, *MLCp* myosin light chain phosphatase, *NO* nitric oxide, *NOS* nitric oxide synthase, *PDE5* phosphodiesterase 5, *PIP3* phosphatidylinositol triphosphate, *PKG* protein kinase G, *PLC* phospholipase C, *sGc* soluble guanylate cyclase, *SMC* smooth muscle cell, *VASP* vasodilatation stimulating proteins. This figure is produced by adapting images from Servier Medical Art by Servier (https://smart.servier.com/), licensed under a Creative Commons attribution 3.0 Unported Licence

The balance between the ET and NO pathways in the pulmonary vasculature under physiological circumstances is nicely illustrated by the data in Healthy swine, where NOSi unmasked a vasodilator effect of subsequent ET_A+B_i. These data suggest that in Healthy swine, the vasoconstrictor effect of ET, mediated through activation of the ET_A_ and/or ET_B_ receptors on vascular smooth muscle cells is balanced by a vasodilator influence, through the endothelial ET_B_ receptor with subsequent activation of the NO-cGMP pathway. NOSi abrogates this vasodilator action of ET_B_-activation, thereby unmasking a vasoconstrictor effect of ET in the Healthy pulmonary vasculature. Conversely, in swine with DM + HC + CKD, the effect of ET_A + B_i was similar in the presence and absence of NOSi, consistent with a loss of endothelial ET_B_ receptors, which was confirmed histologically. Our data imply that an imbalance in the contribution of ET_A_ and ET_B_ to pulmonary vasomotor control—specifically loss of ET_B_-mediated vasodilation—plays an important role in the increase in PVR in our model (Fig. 11). Such protective role of the endothelial ET_B_ receptor against development of PH was also observed in a study in mice with endothelial-specific ET_B_ knockout, which showed aggravated hypoxia-induced PH [28]. Similarly, loss of endothelial ET_B_ receptors in PH was observed in a rat model of monocrotaline-induced PH, in which stimulation of ET_B_ resulted in pulmonary endothelium-dependent pulmonary vasodilation in healthy rats, which was converted into vasoconstriction in rats with PH [27].

Our study suggests that ET-receptor antagonism may be beneficial particularly in early pulmonary vascular disease secondary to multiple comorbidities. Yet, no beneficial effects of 12 weeks of ET_A + B_i with either bosentan or macitentan were observed in patients with HFpEF-PH in the BADDHY [30] and the MELODY-1 [53] studies, respectively, whereas fluid retention did occur as a side-effect of chronic ET_A + B_i in a subgroup of patients in the MELODY-1 trial. However, our model represents early stage disease. Furthermore, as ET-1 levels rise when kidney function declines and endothelin-antagonism may also be reno-protective [29], careful patient selection and/or combination with a diuretic may reveal a subgroup of early HFpEF patients with CKD in which endothelin-antagonism is protective against progression of PVD. Alternatively, other ways of interfering with the NO-ET balance may be beneficial in HFpEF-PH. Relaxin-2, which is a hormone with an insulin-like structure, has been shown to cause NO-mediated vasodilation and interfere with ET-induced vasoconstriction, by upregulating endothelial ET_B_ expression. It also ameliorates endothelial as well as metabolic dysfunction in diabetes, making it a promising therapeutic compound for both HFpEF as well as HFpEF-PH [12, 17]. Future studies should test this interesting compound in our swine model with comorbidities-induced PVD.

## Conclusion

The present study is the first to investigate the effects of three common comorbidities on pulmonary vasomotor control and RV function and structure in swine at rest and during graded treadmill exercise. Our findings demonstrate that, in the absence of overt PH, comorbidities result in increased PVR due to alterations in pulmonary vascular vasomotor control. At this early stage of PVD, neither pronounced pulmonary structural changes nor RV functional and structural changes are present. These findings support the concept that changes in pulmonary vascular vasomotor control are present early in the development of PVD in patients with comorbidities, and suggest that restoring the pulmonary vasomotor balance before overt PH occurs might prove a valuable therapeutic target in patients with early HFpEF and PVD.

## Supplementary Information

Below is the link to the electronic supplementary material.

Supplementary file1 (DOCX 867 KB)
