# Supplementary material for: Impaired pulmonary vasomotor control in exercising swine with multiple comorbidities
Source: Basic Res Cardiol. 2021 Sep 12;116(1):51. doi: 10.1007/s00395-021-00891-7 (PMC8435524; doi:10.1007/s00395-021-00891-7)
Supplement: Supplementary file 1 — Supplementary file1 (DOCX 867 KB) [file 395_2021_891_MOESM1_ESM.docx]

**Supplemental data pertaining to:**

**Impaired Pulmonary Vasomotor Control in Exercising Swine with Multiple Comorbidities**

**Authors:** ^*^Jens van de Wouw^a^ MD, MSc, ^*^Jarno J. Steenhorst^a^ MD, MSc, Oana Sorop^a^ PhD, Ruben W.A. van Drie^a^ BSc, Piotr A. Wielopolski PhD^b^, Alex Kleinjan^c^ PhD, Alexander Hirsch^b,d^ MD PhD, Dirk J. Duncker^a^ MD, PhD and Daphne Merkus^a,e,f^ PhD


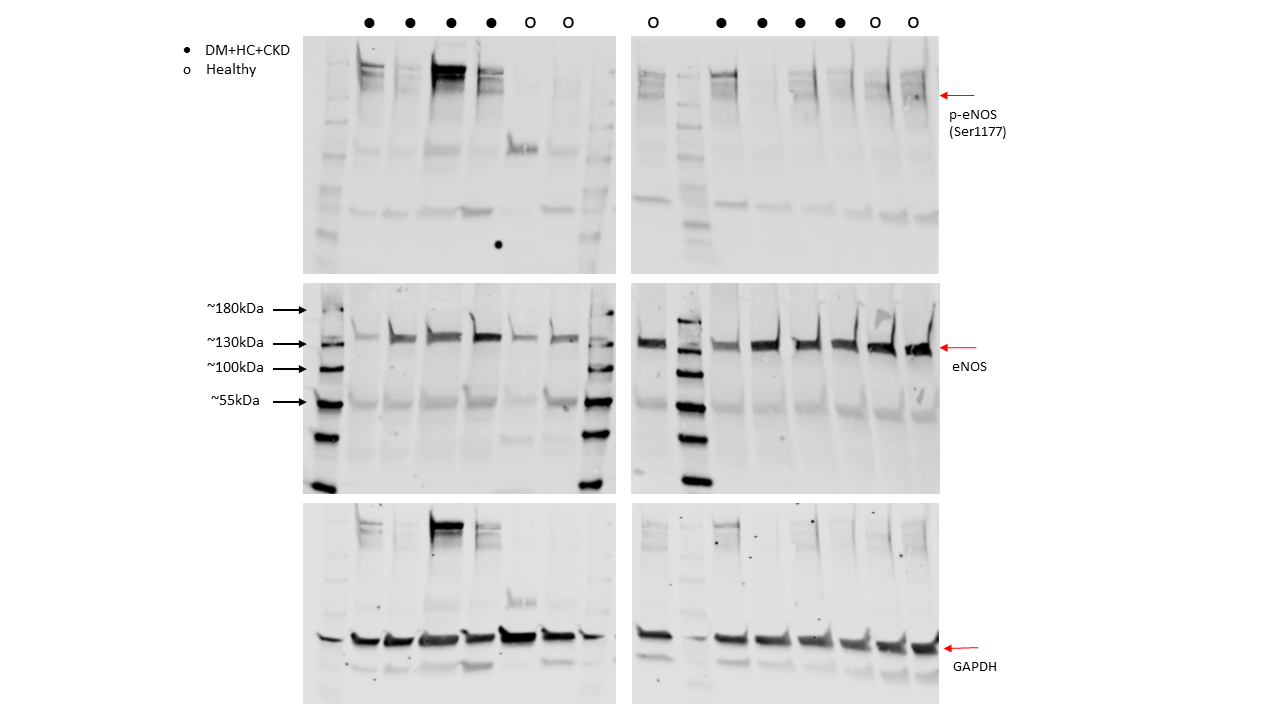


**Supplemental fig. 1: Uncut Western blot of total eNOS protein, phosphorylated eNOS and loading control GAPDH in lung tissue of Healthy and DM+HC+CKD.**


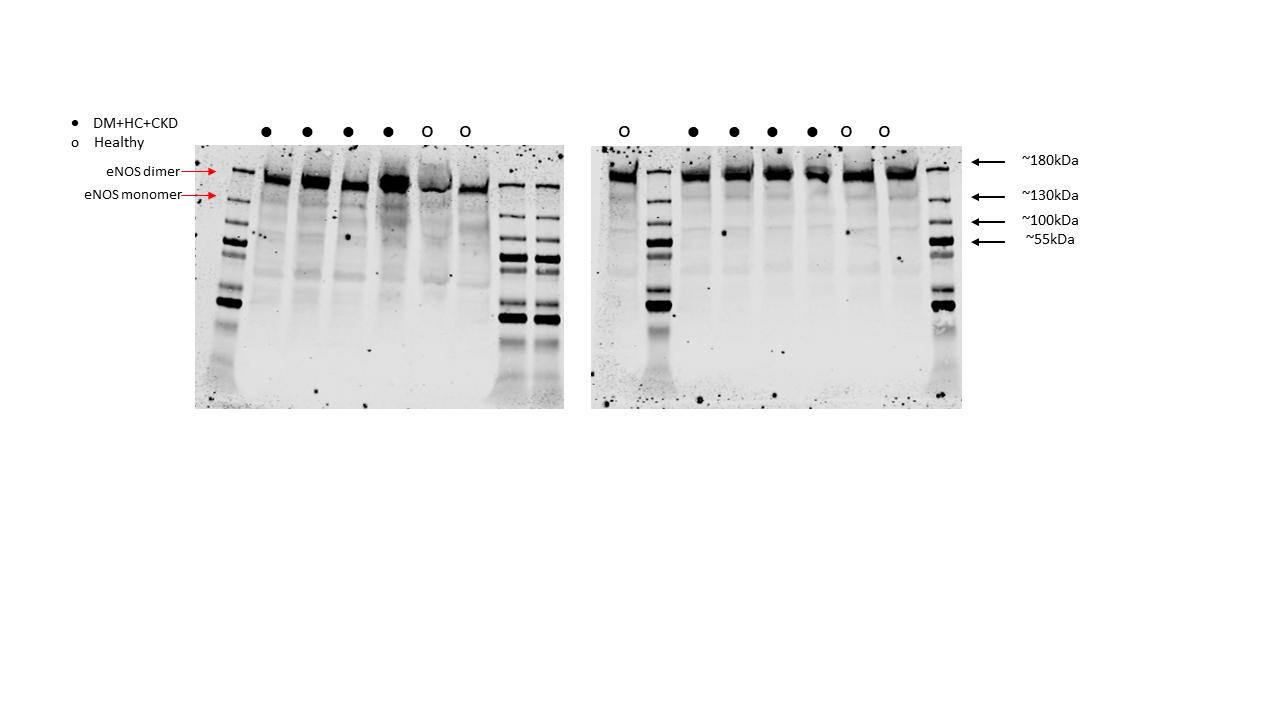


**Supplemental fig. 2: Uncut Western blot of eNOS monomer and dimer in lung tissue of Healthy and DM+HC+CKD.**


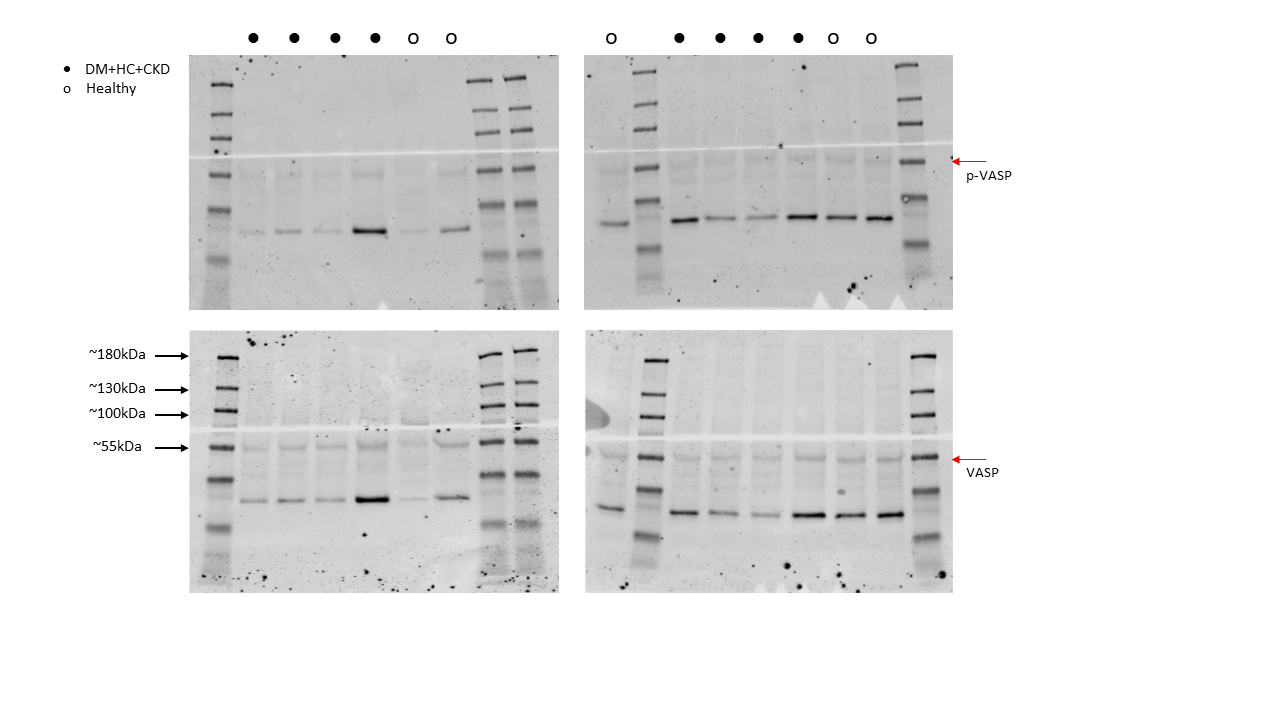


**Supplemental fig. 3: Uncut Western blot of phosphorylated VASP and VASP in lung tissue of Healthy and DM+HC+CKD.** VASP vasodilator stimulated phosphoprotein
